# Nanoparticles induce genetic, biochemical, and ultrastructure variations in *Salvadora persica* callus

**DOI:** 10.1186/s43141-021-00124-3

**Published:** 2021-02-09

**Authors:** Manar S. Fouda, Mohamed H. Hendawey, Ghada A. Hegazi, Hayat M. Sharada, Nagwa I. El-Arabi, Mohamed E. Attia, Elham R. S. Soliman

**Affiliations:** 1 Department of Chemistry, Faculty of Science, Helwan University, Helwan, Cairo, Egypt; 2 Department of Genetic Resources, Desert Research Center, El-Matareya, Cairo, Egypt; 3 Department of Genetics, Faculty of Agriculture, Cairo University, Giza, Egypt; 4 Cytogenetics and Molecular Genetics Unit, Botany and Microbiology Department, Faculty of Science, Helwan University, Helwan, Egypt

**Keywords:** Meswak, Nanoparticles, Benzyl isothiocyanate, ISSR, Antioxidant

## Abstract

**Background:**

*Salvadora persica* is an endangered medicinal plant due to difficulties in its traditional propagation. It is rich in bioactive compounds that possess many pharmaceutical, antimicrobial activities and widely used in folk medicine. The current study aims at in vitro propagation of *Salvadora persica* and the application of different nanoparticles (NPs) to induce the synthesis of bioactive and secondary metabolites within the plant. The cellular and genetic responses to the application of different NPs were evaluated.

**Results:**

The impact of nanoparticles NPs (ZnO, SiO_2_, and Fe_3_O_4_) on callus growth of *Salvadora persica* and the production of its active constituent benzyl isothiocyanate was examined, regarding some oxidative stress markers, antioxidant enzymes, and genetic variabilities. An encouraging impact of 0.5 mg/l ZnO NPs on benzyl isothiocyanate production was shown reaching up to 0.905 mg/g callus fresh weight in comparison to 0.539 mg/g in control callus. This was associated with decreasing hydrogen peroxide content and increasing superoxide dismutase and peroxidase activities. The deposition of the NPs on cellular organelles was detected using a transmission microscope. Fifteen Inter-Simple Sequence Repeats (ISSR) primers detected an overall, 79.1% polymorphism among different treatments. A reduction in genomic DNA template stability (GTS) was made and was more pronounced in higher doses of different NPs.

**Conclusion:**

This study is a stepping stone in developing a productive protocol for in vitro production of benzyl isothiocyanate from *Salvadora persica* using NPs as a valuable anticancer compound.

## Background

Nanotechnology has been extensively employed in plant biotechnology and has monumental applications. In agriculture, it is related to nutrient utilization, plant germination, plant development, and stress tolerance. The application of nanoparticles (NPs) to induce the synthesis of bioactive secondary metabolites from their natural sources is a rising trend. Their potential tiny size, wide surface area, reactivity, and high affinity to penetrate the plasma membrane make them ideal for biological applications, taking into consideration their various effects on living cells [1]. However, the role of NPs in plant secondary metabolism regarding callus culture continues to be obscure [2]. Several studies reported the positive influence of NP application on plant growth and development, particularly at low concentrations. However, a degree of phytotoxic impact of NPs, particularly at high concentrations, was conjointly reportable. A promotive effect on *Salvadora persica* callus growth and benzyl isothiocyanate accumulation was observed after callus exposure to TiO_2_ and CuO NPs at concentrations of 0.5–2 mg/l [1]. *Arabidopsis thaliana* (L.) Heynh seedlings treated with 200 and 300 mg/l ZnO NPs demonstrate reduced, growth, chlorophyll content, and rates of photosynthesis [2]. A similar impact was discovered in *Oryza sativa* treated with 1000 mg/l CuO NPs [3]. Callus growth of rapeseed was significantly retarded by 25, 50, and 100 mg/l ZnO NPs, while 10 mg/l significantly induce callus growth [4].

The tissue culture technique is widely used for sustainable conservation and utilization of valuable secondary metabolites in rare and endangered medicinal plants, particularly those with difficulties in their traditional propagation, such as *Salvadora persica* L. [5]. *Salvadora persica* is commonly known as Meswak (toothbrush) and belongs to the family Salvadoraceae*.* Traditionally, the plant is employed in folk medicine for oral hygiene, dental care, cough, asthma, scurvy, rheumatism, ulcers, and piles curing. *Salvadora persica* contains several bioactive molecules, such as volatile oils, flavonoids, alkaloids, terpenoids, and saponins in different parts of the plant. These constituents have many pharmacological activities and antimicrobial properties [6]. *Salvadora persica* aqueous root extract exhibits cytotoxicity on oral cancer cells: oral epithelial dysplasia (DOK) and oral squamous cell carcinoma (PE/CA-PJ15) [7]. Also, the cytotoxicity of *Salvadora persica* sticks and bark extracts against HepG2, MCF7, A549, and HCT116 cancer cells was investigated. Cytotoxic properties of the plant extract were investigated against the breast MCF7, ovary A2780, and colon HT29 cells. The fruit extract of *Salvadora persica* was selective against the ovarian and colon cancer cells [8]. Besides the various properties of *Salvadora persica*, ZnO NPs can be synthesized using the root extract of the plant from its precursor, zinc acetate dihydrate. The existing volatiles, essential oils, alkaloids, and acids in the root extract help in ZnO NP fabrication. These NPs can be used in medical, photochemical, biosensors, and electrical applications [9].

*Salvadora persica* is a cross-pollinated tree with a high degree of genetic variability and low seed formation and viability (30%) and needs 7–9 years for complete maturation. All of these restrict its propagation. In vitro propagation of *Slavadora persica* offers an associate economical route for the production of virus-free plantlets in a faster time [10].

*Salvadora persica* is a reliable natural supply for extracting benzyl isothiocyanate (BITC) that is believed to be promising within the development of anticancer medications. Recently, BITC found to inhibit prostate cancer [11], lung cancer [12], oral cancer [13], and invasion of hepatocellular carcinoma [14].

Nanoparticle utilization by plant cells can cause genetic variations that are species, NP size, type, and concentration-dependent. Very little is thought concerning the genotoxicity of NPs in plants; therefore, far more effort should be done for evaluating plant genetic response [15]. To achieve this goal, several DNA-based techniques, such as Inter Simple Sequence Repeats (ISSR) and Random Amplification of Polymorphic DNA (RAPD) techniques, have been with efficiency accustomed to assess the genotoxic impact of NPs on various plant species [16–18]. The generation of ISSR markers makes use of microsatellite sequences that are highly variable and ubiquitously distributed across the genome. All these make ISSR an ideal genetic marker for genetic variation [19, 20], DNA fingerprinting [21], and genotoxicity analysis [18].

The present study was aimed toward evaluating the potential impact of various concentrations of three NPs; Fe_3_O_4_, SiO_2_, and ZnO on *Salvadora persica* callus. The evaluation includes callus biomass, BITC production, antioxidant enzyme activities, cellular response, and genetic variations.

## Methods

### *Salvadora persica* callus induction and nanoparticles treatment

*Salvadora persica* shoots bearing eight to ten nodes with terminal buds were excised from 30-day-old uniform plants derived from seeds obtained from the Red Sea coastal region. Experiments were carried out at the Desert Research Center (DRC), Cairo, Egypt. The plant was identified by Dr. Omran Ghaly, Head of Plant Taxonomy Unit, Desert Research Center, Egypt, given the voucher number CAIH-1008-R, and the voucher specimen was deposited in the Herbarium of Desert Research Center (CAIH).

Stem internodal segment explants were cut and washed under running tap water for 15 min with 1% of a commercial detergent (Pril). Surface sterilization of the explants in a laminar airflow hood (Holten LaminAir HVR 2448, USA) by submerging the explants in 30% of commercial bleach (Clorox containing 5.25% sodium hypochlorite) for 10 min, followed by dipping in 0.1% (w/v) mercuric chloride (HgCl_2_) for 45 s. Finally, explants were thoroughly rinsed five times with sterilized distilled water.

*Salvadora persica* callus was induced from stem internodal segments on MS medium [22] (Duchefa, Haarlem, the Netherlands), supplemented with 3% (w v^-1^) sucrose, 1 mg/l 2,4-dichlorophenoxyacetic acid (2,4-D), and 1 mg/l kinetin (kn) (Sigma Cell Culture, min. 90%, St. Louis, USA) according to Hegazi et al. [23]. The pH of the medium was adjusted to 5.7–5.8 and solidified with 0.25% (w v^-1^) phytagel (Duchefa, Haarlem, the Netherlands), before autoclaving at a pressure of 1.06 kg cm^-2^ and 121 °C for 15 min. Four explants were cultured per jar, and the cultures were incubated at 25 ± 2 °C in the darkness and sub-cultured every 3 weeks.

Pure preparation (99%) of ZnO, SiO_2_, or Fe_3_O_4_ nanopowder, 25 to 55 nm in size, was purchased from Sigma-Aldrich (Germany). The NPs were dissolved in sterilized MS medium to final concentrations of 0.5 or 2 mg/l of ZnO, SiO_2_, or Fe_3_O_4_. Callus fresh weight (CFW) was recorded after 30 and 45 days of incubation.

### Extraction and HPLC analysis of benzyl isothiocyanate

Benzyl isothiocyanate content was determined by high-performance liquid chromatography (HPLC) (Dionex Ultimate 3000 equipped) according to Kiddle et al. [24]. Briefly, 3 g of 30- or 45-day NP-treated or untreated callus were homogenized with 5 ml of methanol and deionized water 70: 30 (v/v) at 70 ^o^C for 30 min. The extracts were centrifuged at 8000 rpm for 20 min. The supernatants were filtered with a 0.45-mm membrane filter. The HPLC was performed by UV-VIS detector at 246 nm wavelength, an injection volume of 20 μl at 40 °C, C18 column (250 × 4.6 mm), mobile phase acetonitrile and deionized water (60: 40 v/v), and flow rate of 1 ml/min. Four standard concentrations of BITC (2000, 1000, 500, and 250 mg/l) (Sigma-Aldrich Germany) were separately analyzed by HPLC. The standard curve was plotted between concentration and peak area, and the best line was chosen. Benzyl isothiocyanate content in callus extracts, expressed as mg/g CFW, was calculated by using the equation of the standard curve.

$$ Y=12.125X+0.0207 $$

### Oxidative stress

Malondialdehyde and hydrogen peroxide (H_2_O_2_) contents were determined as markers for oxidative stress. Also, the activity of some reactive oxygen species scavenging enzymes; superoxide dismutase and peroxidase were detected in 45-days-old NP-treated callus in comparison to control.

#### Malondialdehyde content

*Malondialdehyde* (MDA) level was determined according to Heath and Packer [23]. Three grams of fresh weight of callus of NP treatments and the control treatment were homogenized in 1 ml of 0.1% trichloroacetic acid (TCA) (w/v) and then centrifuged at 14,000 rpm for 5 min. Two ml of thiobarbituric acid (TBA) reagent was added to 0.5 ml of the supernatant. The mixture was heated at 95 ^o^C for 15 min and cooled immediately. MDA-TBA complex concentration was determined spectrophotometrically (Spectronic Genesys. 5) at 532 nm and corrected by subtracting the absorbance at 600 nm and converted to μmol/g CFW (calculated from MDA standard curve).

#### Hydrogen peroxide content

Hydrogen peroxide content was determined according to Junglee et al. [25]. Three grams of fresh weight of NP-treated and control callus were homogenized in 1 ml 0.1% TCA (w/v), then centrifuged at 10,000 rpm for 15 min. Half milliliter of each supernatant was added to 0.5 ml of 10 mM potassium phosphate buffer (pH 7.0) and 1 ml of 1 M KI. The absorbance was measured at 390 nm spectrophotometer (Spectronic Genesys. 5). The content of H_2_O_2_ in *Salvadora persica* callus was converted from mg/l (calculated from H_2_O_2_ standard curve) to μmol/g CFW.

#### Antioxidant enzymes

Three grams of fresh weight of *Salvadora persica* callus were homogenized in 2 ml extraction buffer (0.61 g Tris-HCl pH 7.5, 5% (v/v) glycerol, 14 mM β-mercaptoethanol 0.1% (v/v), followed by centrifugation at 8000 rpm for 15 min at 2 ^o^C. Protein concentration was measured in each extract using Bradford methods (Bio-Rad, CA), and bovine serum albumin (BSA) was used as a standard. Thirty micrograms of protein extract were mixed with non-reducing sample loading buffer (62.2 mM Tris-HCl pH 6.8, 10% (v/v) glycerol, 1% (w/v) bromophenol blue without SDS) and resolved on 10% polyacrylamide gel. After electrophoresis, superoxide dismutases (SOD) activity was visualized in native polyacrylamide gel electrophoresis (PAGE) by incubating the gel in 0.2 M Tris-HCl (pH 8.0) containing 4% riboflavin, 4% EDTA, and 20% nitro blue tetrazolium **(**NBT) for 40 min in dark [26]. The SOD activity appeared as a zone of clearance on a blue background. Peroxidase activity (POD) was visualized in native PAGE by staining the gels in 0.2 M acetate buffer (pH 4.8) containing 3% H_2_O_2_ and 4% 3,3′,5,5′-tetramethylbenzidine (TMB) in 50% methanol at room temperature, till the yellow color appeared [27]. Band intensities were quantified using Quantity One software.

### Genetic variation and ISSR profiling

The ISSR analysis was applied to evaluate the effect of NPs on genetic contents of 45-day-old *Salvadora persica* callus treated with 0.5 or 2 mg/l of Fe_3_O_4_, SiO_2_, or ZnO NPs in comparison to non-treated callus; control. The total genomic DNA was isolated from all samples according to the developed protocol of Porebski et al. [28] for plants containing high polysaccharides [28]. For ISSR analysis, the polymerase chain reaction was carried out in Biometra thermal cycler using primers in 50-μl reaction volume. The PCR reaction mixture included the following: 30 ng of DNA, 0.5 U of Red Hot *Taq* polymerase (AB-gene House, UK), and 1X *Taq* polymerase buffer (AB-gene House, UK), 10 mM dNTPs, 50 mM MgCl_2_, and 10 μM of each primer. The PCR profile starts with 94 °C for 5 min, followed by 38 cycles of denaturation at 94 °C for 1 min, annealing temperature as shown in Table 4 for 1 min and extension at 72 °C for 2 min. A final extension at 72 °C for 10 min was included. 1.5% (w/v) agarose gel in 1X TAE buffer containing 0.5 μg/ml ethidium bromide was used to resolve the PCR products. Ethidium bromide-stained gel was visualized and image captured using UV-transilluminator. The bands (ISSR markers) were scored using Quantity One software version 4.6.2.70. The band occurrence was scored as binary data; 1 for the presence and 0 for the absence. The number of unique, polymorphic, and monomorphic bands and the percentage of polymorphism for each primer and each treatment were calculated. GeneRuler 1 kb DNA ladder (Thermo scientific #SM1331) was used to determine the size of the ISSR fragments.

The percent of genomic template stability (% of GTS) was calculated according to the following formula:

$$ \%\mathrm{of}\ \mathrm{GTS}=\left(1-\frac{a}{n}\right)x100 $$

where *a* is the average number of changes in each DNA profile of each treatment and *n* is the number of total bands in control samples [16].

### Transmission electron microscopy

Control and NP-treated *Salvadora persica* callus cells were imaged by transmission electron microscopy (TEM) to observe the NP deposition in the callus cells. Samples were washed and fixed in 0.1 M sodium cacodylate buffer (pH 7.0) containing 3% glutaraldehyde for 2 h at room temperature, then post-fixed in 1% osmium tetraoxide for another 2 h at room temperature. Next, the samples were immersed in gradient ethanol for dehydration from 10 to 90% for 15 min in each alcohol dilution and finally dehydrated with absolute ethanol for 30 min. The samples were infiltrated with epoxy resin and acetone through a graded series till in pure resin. Ultrathin sections were collected on former-coated copper grids. Sections were then double-stained in uranyl acetate, followed by lead citrate. Stained ultrathin sections of samples were examined with a JEOL 1010 TEM at 70 kV [29].

### Experimental design and statistical analysis

Experiments were subjected to a completely randomized design. Analysis of variance (ANOVA) was used to analyze data using Duncan’s multiple range test [30] as modified by [31] to compare the means at *p* < 0.05.

## Results

### Effect of nanoparticles on callus biomass

The results of the present study showed that the treatment of *Salvadora persica* callus with different concentrations of ZnO, SiO_2_, and Fe_3_O_4_ NPs had positive effects compared with control treatment, during the two growth stages. The increase in callus fresh weight was the highest in callus treated with 2 mg/l of ZnO NPs or SiO_2_ NPs for 45 days, reached up to 256 and 250% increase over the control callus, respectively as shown in Table 1.

**Table 1:** Effect of nanoparticles on fresh weight of *Salvadora persica* callus after 30 and 45 days of treatment

Type of NPs	NP treatments (mg/l)	Callus at 30 days old			Callus at 45 days old		
		Fresh weight (g/jar) *M* ± SD	*P.* value	% of increase in fresh weight	Fresh weight (g/jar) *M* ± SD	*P* value	% of increase in fresh weight
	Control	1.56 ± 0.03			2.61 ± 0.36		
Fe_3_O_4_	0.5	* 2.27 ± 0.21	0.042	71	3.27 ± 0.31	0.073	66
	2	* 3.43 ± 0.21	0.002	187	* 4.21 ± 0.53	0.016	160
SiO_2_	0.5	1.73 ± 0.21	0.499	17	2.63 ± 0.51	0.932	2
	2.0	* 3.63 ± 0.21	0.001	207	* 5.17 ± 0.53	0.001	250
ZnO	0.5	* 3.83 ± 0.65	0.013	227	* 4.93 ± 0.70	0.015	232
	2.0	* 3.91 ± 0.26	0.001	235	* 5.17 ± 0.35	0.001	256

*P* value ≤ 0.05 * is significant. *M* + SD mean ± standard deviation

### Effect of nanoparticles on benzyl isothiocyanate content

Benzyl isothiocyanate content in NP treated and untreated callus for 30 and 45 days was detected at 246 nm based on UV–Visible spectral data. The HPLC profile of BITC standard showed the presence of one peak with a retention time of 2.767 min. HPLC analysis showed that the maximum accumulation of BITC (~0.91 mg/g) was recorded at 0.5 mg/l ZnO NP callus treatment for 45 days, comparing to all applied NPs. Benzyl isothiocyanate content slightly increased in callus treated with 2 mg/l of SiO_2_ or Fe_3_O_4_ NPs for 45 days compared with control as illustrated in Table 2.

**Table 2:** Effect of nanoparticles on benzyl isothiocyanate content in *Salvadora persica* callus after 30 and 45 days of treatment

	BITC content (mg/g callus fresh weight)						
		30 days of callus growth			45 days of callus growth		
Type of NPs	NPs treatments (mg/l)	Area under curve	Retention time (min)	BITC content (mg/g)	Area under curve	Retention time (min)	BITC content (mg/g)
Control		127.188	2.763	0.514	133.357	2.760	0.539
Fe_3_O_4_	0.5	115.097	2.767	0.465	113.801	2.753	0.461
	2.0	136.305	2.787	0.551	143.540	2.767	0.581
SiO_2_	0.5	106.914	2.777	0.432	102.455	2.747	0.414
	2.0	115.477	2.763	0.467	163.535	2.767	0.661
ZnO	0.5	128.735	2.780	0.521	223.844	2.810	0.905
	2.0	146.525	2.757	0.592	127.360	2.780	0.515
		Area under curve	Retention time (min)	BITC content (mg/g)			
Mother plant		73.147	2.787	0.296			

### Effect of nanoparticles on oxidative stress

#### Altered malondialdehyde and hydrogen peroxide levels in nanoparticle-treated callus

To evaluate the oxidative stress and lipid peroxidation induced by ZnO, SiO_2_, and Fe_3_O_4_ NP treatments in *Salvadora persica* callus after 45 days, the levels of MDA and H_2_O_2_ in callus homogenate were estimated. Table 3 shows a significant increase of MDA levels after treatment with Fe_3_O_4_, SiO_2_, or ZnO NPs with different concentrations, compared with the control, except 0.5 mg/l ZnO NPs that had a non-significant effect on MDA, whereas callus treated with all NP treatments recorded a significant decrease in H_2_O_2_ level, except for 2.0 mg/l Fe_3_O_4_ NP treatment.

**Table 3:** Effect of nanoparticles on MDA and H_2_O_2_ levels in *Salvadora persica* callus after 45 days of treatment

	Lipid peroxidation				
	NP treatments (mg/l)	MDA level (μmol/g CFW) M ± SD	*P.* value	H_2_O_2_ level (μmol/g CFW) *M* ± SD	*P.* value
	Control	43.73 ± 0.75		0.867 ± 0.05	
Fe_3_O_4_	0.5	* 55.75 ± 3.75	0.020	* 0.260 ± 0.03	0.001
	2.0	* 122.80 ± 4.16	0.001	0.984 ± 0.11	0.193
SiO_2_	0.5	* 58.83 ± 4.25	0.020	* 0.517 ± 0.01	0.005
	2.0	*68.83 ± 4.16	0.010	* 0.417 ± 0.01	0.003
ZnO	0.5	43.25 ± 2.25	0.752	* 0.501 ± 0.03	0.001
	2.0	* 54.17 ± 3.51	0.008	* 0.401 ± 0.05	0.001

*P* value ≤ 0.05 * is significant. *M* + SD: mean ± standard deviation

*MDA* malondialdehyde, *CFW* callus fresh weight

#### Altered superoxide dismutases and peroxidase activities in nanoparticle-treated callus

**Fig. 1:**
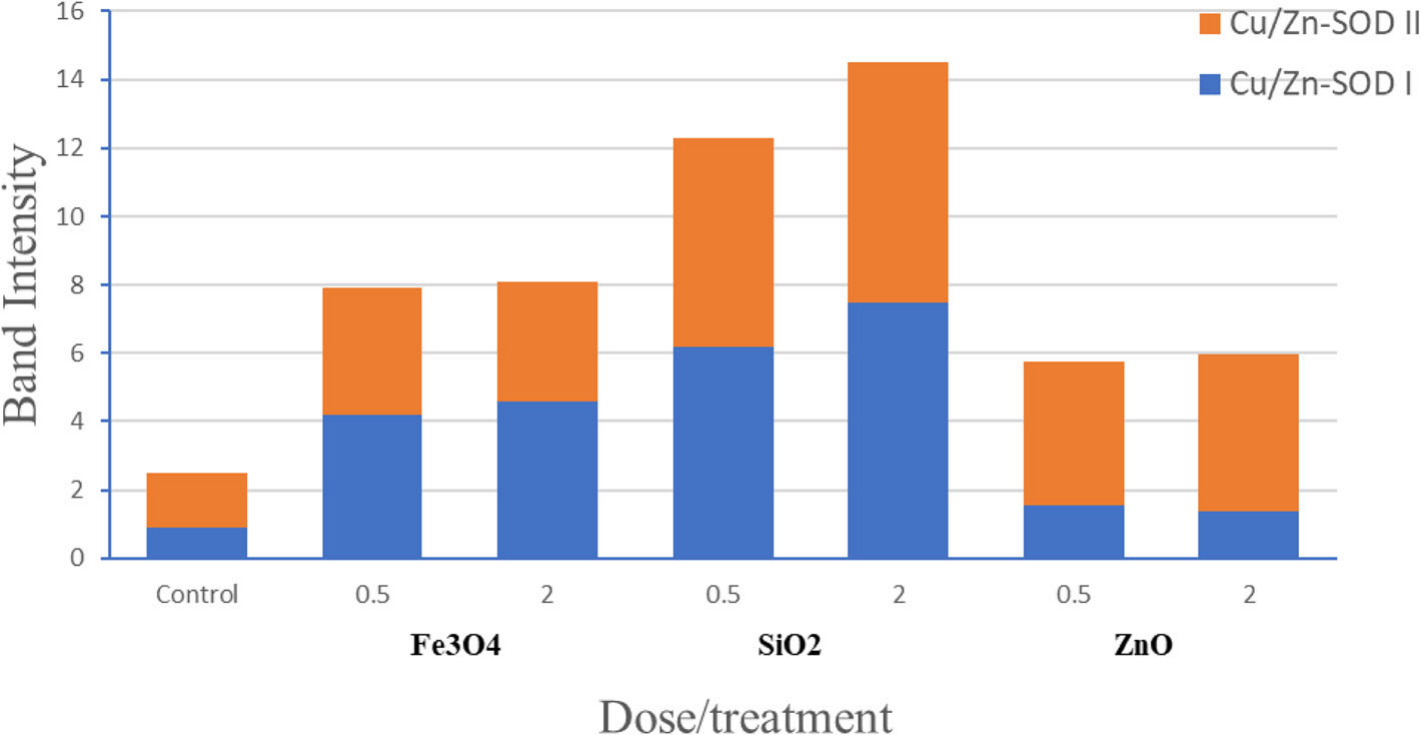
Activity of SOD isoforms in 45 days old *Salvadora persica* callus treated with Fe_3_O_4_, SiO_2_, or ZnO nanoparticles and the control. The SOD activity was represented as relative band intensities of Cu/ZnSODI and Cu/ZnSODII

The SOD and POD activities were determined by in-gel assays using proteins isolated from *Salvadora persica* callus treated with Fe_3_O_4,_ SiO_2_, and ZnO NPs. Five main bands showing SOD activity were detected, one band for Mn SOD, two bands showing FeSOD activity (FeSODI and FeSODII), and two bands showing Cu/ZnSOD activity (Cu/ZnSODI and Cu/ZnSODII). No consistent differences were observed in MnSOD, but different isoforms of FeSOD activity between control and NP-treated callus were observed, although an increase in the activity of Cu/ZnSOD isoforms was detected in NP-treated callus vs. control (Fig. 1).

**Fig. 2:**
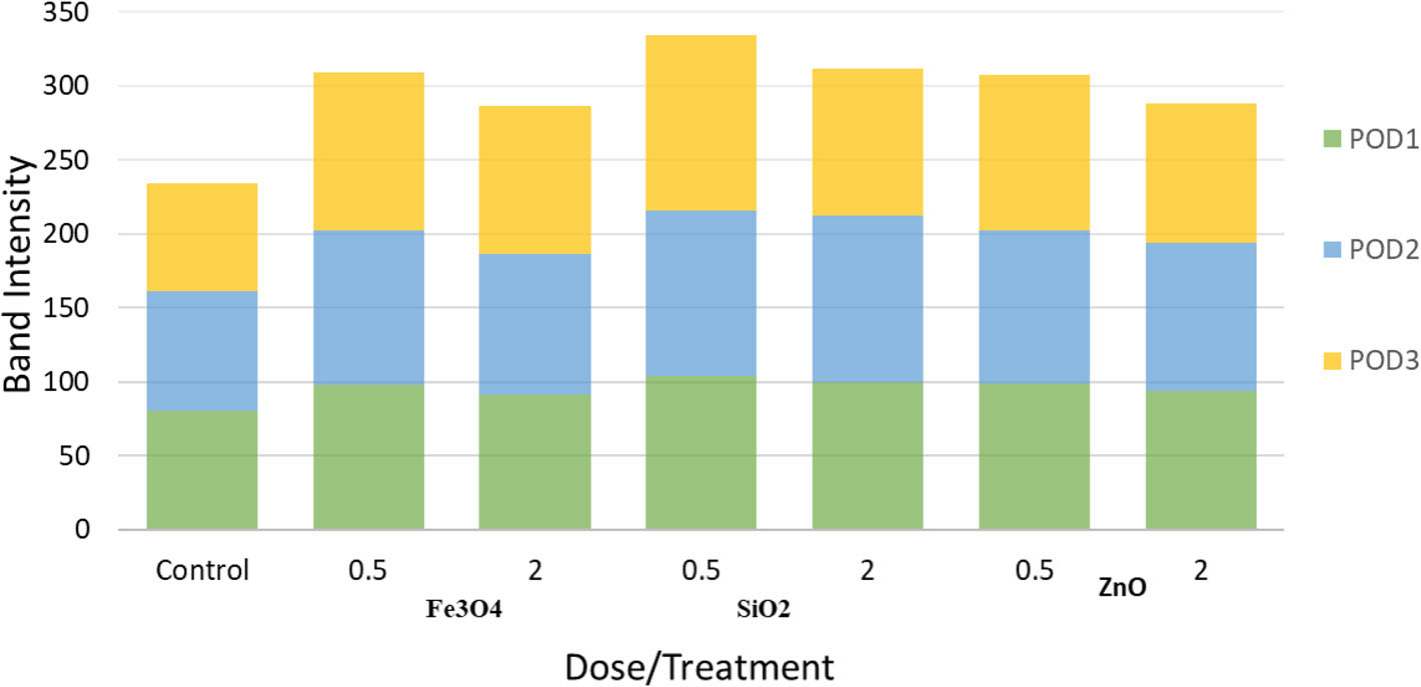
Activity of POD isoforms in 45 days old *Salvadora persica* callus treated with Fe_3_O_4_, SiO_2_, or ZnO nanoparticles and the control. The POD activity was represented as relative band intensities of PODI, PODII, and PODIII

Three main bands showing different POD isoform (POD1-POD2 and POD3) activity were detected. An increase in all POD isoforms in NP-treated callus compared with control was detected, in which POD isoform activity in callus treated with Fe_3_O_4_ and SiO_2_ NPs was higher than that of ZnO NPs treatments (Fig. 2).

### Genetic variation analysis induced by nanoparticles

**Fig. 3:**
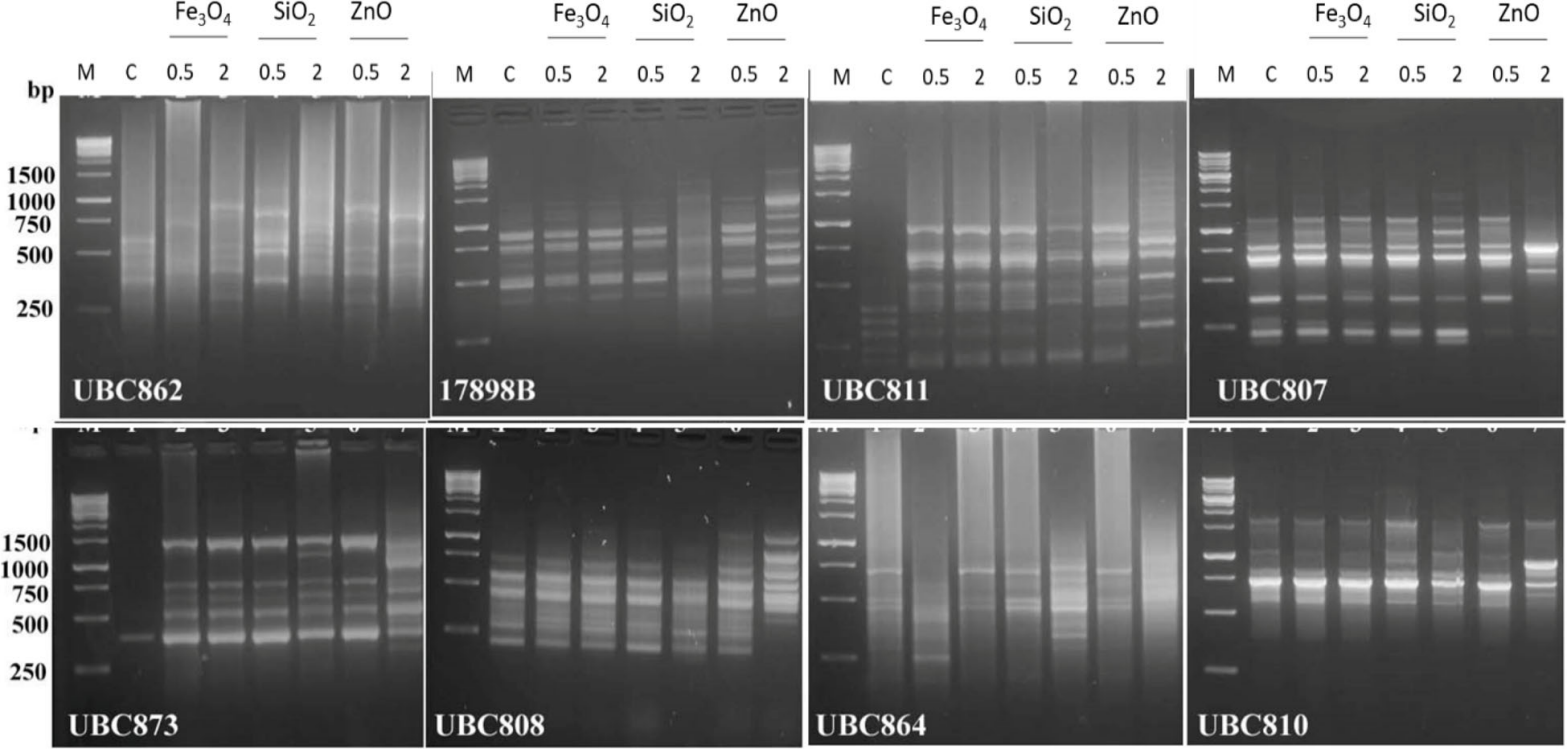
ISSR profiles produced from *Salvadora persica* callus. The ISSR profile produced in 45-day-old callus treated with 0.5 and 2 mg/l of Fe_3_O_4_, SiO_2_, or ZnO nanoparticles and their controls (C). The ISSR profile produced using eight different primers as labeled on each gel image. M: 1 kb DNA ladder

Analysis of ISSR was applied to investigate the NP effect on the genetic contents of 45-day-old *Salvadora persica* callus subjected to different treatments of NPs. Fifteen ISSR primers were successfully detected 191 alleles among different treatments. The overall primers revealed 151 polymorphic markers representing 79.1% of polymorphism. The most reproducible one was UBC811 that scored 23 markers. Nevertheless, UBC826 scored 100% of polymorphism, while UBC862 and UBC824 scored the lowest percentage of 37.5% polymorphism (Table 4 and Fig. 3).

**Table 4:** List of selected ISSR primers and their codes, sequences, annealing temperature, the number of amplified markers, and percentage of polymorphism for each primer in *Salvadora persica* samples treated with Fe_3_O_4_, SiO_2_, or ZnO nanoparticles and their controls

No.	Primer code	Sequencing (5′-3′)	Annealing Temp.	Total band	Monomorphic band	Polymorphic band	Unique		Polymorphism%
							+ve	-ve	
1	17898A	(CA)_6_AC	49	16	6	10	1	2	62.5
2	17899A	(CA)_6_AG	49	12	4	8	3	2	66.6
3	17898B	(CA)_6_GT	50	16	2	14	7	7	87.5
4	17899B	(CA)_6_GG	50	8	4	4	0	1	50
5	UBC807	(AG)_8_GT	52	14	2	12	5	4	85.7
6	UBC808	(AG)_8_GC	52	11	3	8	1	3	72.7
7	UBC810	(GA)_8_T	52	10	3	7	4	2	70
8	UBC811	(GA)_8_AC	52	23	1	22	7	5	95.6
9	UBC814	(CT)_8_A	52	12	1	11	0	3	91.6
10	UBC824	(TC)_8_G	52	8	5	3	0	0	37.5
11	UBC826	(AC)_8_C	52	13	0	13	2	0	100
12	UBC828	(TG)_8_A	52	15	2	13	2	0	86.6
13	UBC862	(AGC)_6_	52	8	5	3	1	0	37.5
14	UBC864	(ATG)_6_	52	14	1	13	5	1	92.8
15	UBC873	(GACA)_4_	52	11	1	10	6	2	90.9
	**Total**			**191**	**40**	**151**	**44**	**32**	**79.1**

**Fig. 4:**
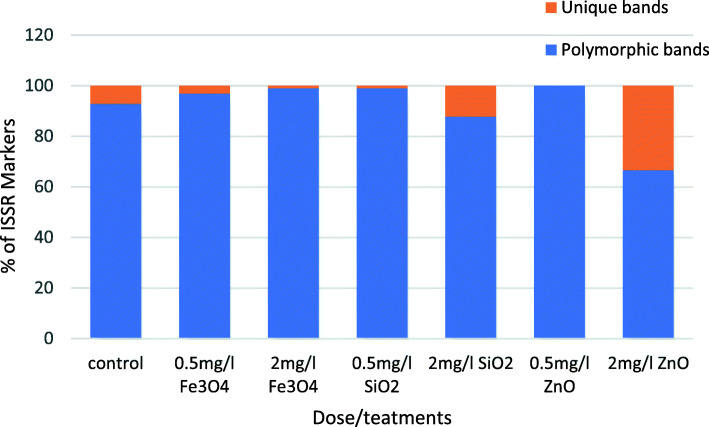
Percentage of polymorphic and unique ISSR markers in *Salvadora persica* callus in 0.5 and 2 mg/l of Fe_3_O_4_, SiO_2_, or ZnO-treated callus of 45 days old and the control

The 2 mg/l dose of both SiO_2_ and ZnO NPs produced the highest percentage of unique markers of 12 and 33%, respectively. On the contrary, the lowest doses of the same NPs generated only one and no specific markers, respectively (Fig. 4).

**Fig. 5:**
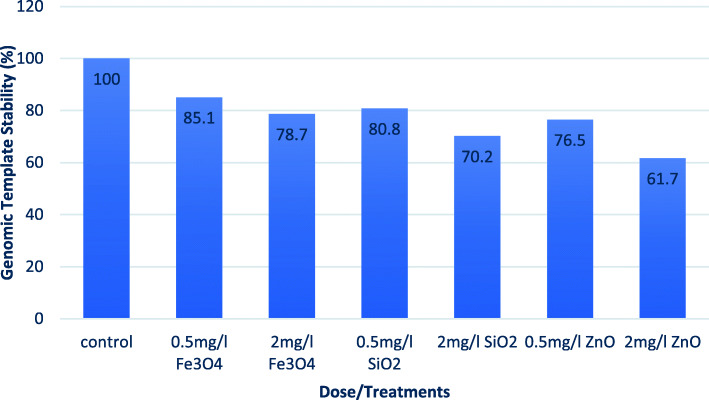
Comparison of genomic DNA template stability (GTS) in 45-day-old callus treated with 0.5 and 2 mg/l of Fe_3_O_4_, SiO_2_, or ZnO nanoparticles and the control in *Salvadora persica* callus

Diversity in the ISSR profile generated by different concentrations of each NP was calculated as a quantitative measure reflecting the percentage of genomic template stability (GTS) for each treatment (Fig. 5). The percentage of GTS was generally decreased in all treatments. The highest GTS was 85.1% for 0.5 mg/l Fe_3_O_4_, while the lowest was 61.7% for 2 mg/l ZnO treatment.

### Visualization of nanoparticles in callus by transmission electron microscope

**Fig. 6:**
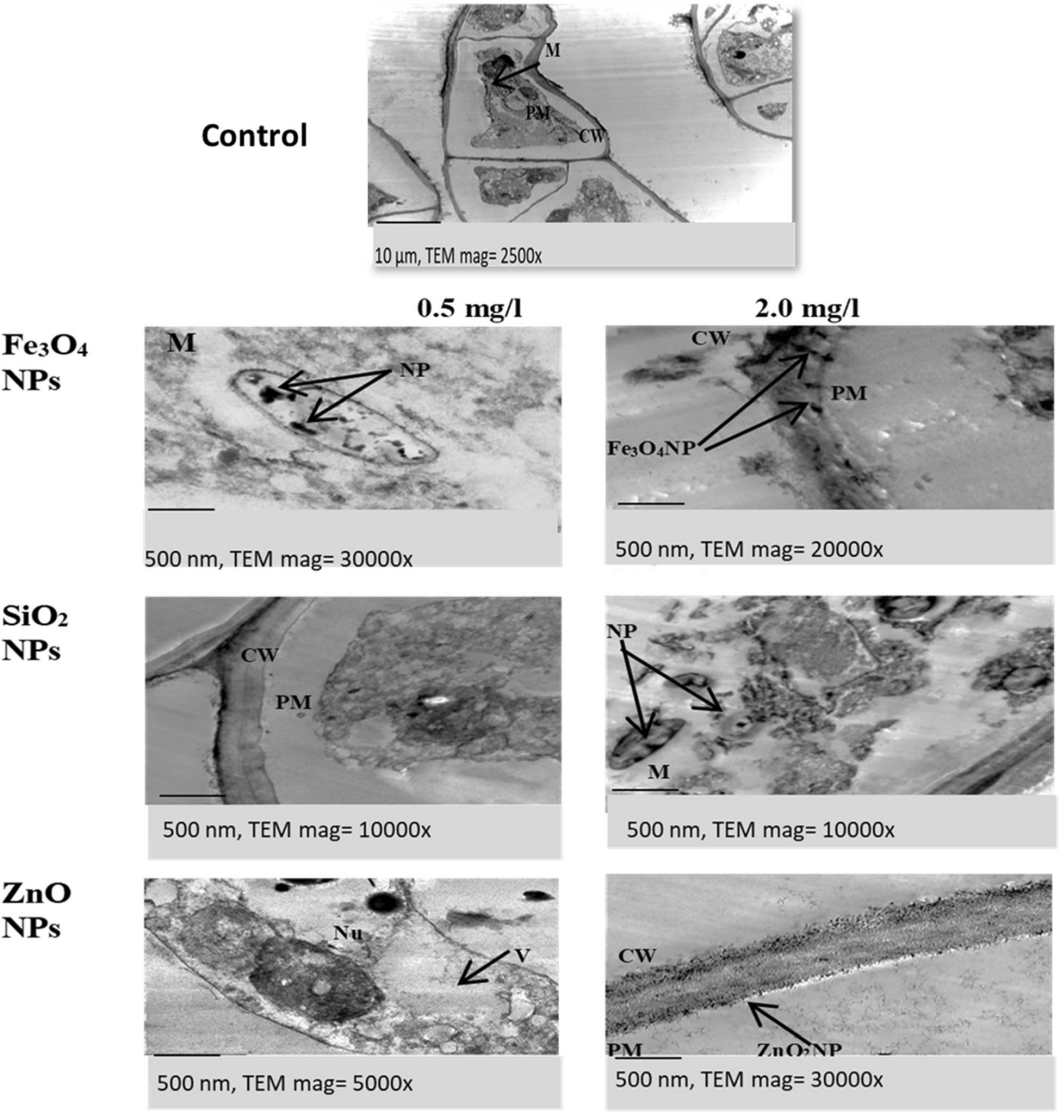
TEM images of 45-day-old callus of *Salvadora persica* treated with 0.5 and 2 mg/l of Fe_3_O_4_, SiO_2_, or ZnO nanoparticles and the control. On each image, the scale bar and the magnification power. Abbreviations on images: CW cell wall, M mitochondria, NP nanoparticle, Nu nucleus, P plasma membrane, V vacuole

Transmission electron microscope images of the cross-callus sections in Fig. 6 show a normal cellular organization in the control callus section. Images of ZnO NP-treated callus show maintained cell components, with the complete cell membrane and cell wall at 0.5 mg/l ZnO or SiO_2_ NPs, while 2 mg/l of both NPs showed accumulation of NPs in the plasma membrane. Treatment with 0.5 mg/l of Fe_3_O_4_ NPs showed accumulation of NPs inside the mitochondria and plasma membrane, which was more pronounced at 2 mg/l NP treatments, which appeared as dark dots.

## Discussion

Nanoparticles are recently used to improve plant growth, yield, and the production of bioactive compounds [16, 32]. In plant cell culture approaches, callus culture was a suitable method for the production of bioactive compounds [33]. *Salvadora persica* is a valuable medicinal plant for the production of bioactive secondary metabolite content such as BITC [23], which has a potent anti-tumor activity [11, 14]. In the current study, Fe_3_O_4_, SiO_2_, and ZnO NPs of 0.5 or 2 mg/l concentrations were applied to *Salvadora persica* callus for two growth stages of 30 and 45 days, to evaluate the effect of NPs on callus biomass and to improve BITC production. Nanoparticle application showed positive effects on the fresh weight of *Salvadora persica* callus during the two growth stages, and this effect was NP type and concentration-dependent. The concentration of 2 mg/l for all applied NPs was significantly the most effective. Several studies have shown significant effects of NPs on callus induction and growth. Application of 10 mg/l of ZnO NPs can induce callus and shoot regeneration in rapeseed, in contrast, callus growth is significantly retarded by 25, 50, and 100 mg/l ZnO NPs [34]. In another study, Javed et al. [35] reported that 1 and 10 mg/l ZnO NPs are the best concentrations regarding the fresh weight of *Stevia rebaudiana* Bertoni callus in comparison with control. The concentration of 1.5 mg/l of Fe_3_O_4_ strongly induces callus size of flax [36]. It can be explained by the proven fact that optimum concentration of different NPs may act as abiotic elicitors for improving callus growth, increasing chlorophyll level and cell division that really reflects on plant fresh weight [35, 36].

The BITC content in *Salvadora persica* callus was variously affected according to NP type, concentration, and callus growth stage, in which 0.5 mg/l ZnO NPs greatly enhanced BITC contents of 45-day-old callus. On the other hand, BITC accumulation slightly increased in callus treated with 2 mg/l SiO_2_ NPs for 45 days, while no changes were observed in 30-day-old callus treated with the same NP concentration. Similar findings were reported for the accumulation of thymol and carvacrol in thyme callus stressed with different concentrations of ZnO NPs [37].

By comparing the growth of callus with the accumulation of BITC, it was observed that due to the application of NPs at different concentrations, Fe_3_O_4_ NPs at both concentrations and SiO_2_ at 0.5 mg/l decreased the callus growth and secondary metabolism, as a nonessential mechanism of plant life, also decreased by increasing exposure time, while SiO_2_ NPs at 2 mg/l and ZnO NPs stimulated callus growth. They greatly increased the accumulation of BITC, except ZnO NPs at 2 mg/l that may be a supra-optimum concentration that inhibited the accumulation of BITC after prolonged exposure (45 days). The synthesis of secondary metabolites in plants is closely related to the growth stage and environmental conditions, and each type of secondary metabolites has different biosynthetic pathways at different stages of growth. In general, the secondary metabolite concentration peaked during the exponential growth stage and decreased afterward during the stationary stage of the callus culture [38].

The NPs can elicit oxidative damage in plants by generating reactive oxygen species (ROS), such as H_2_O_2_ and hydroxyl radical, causing damage to lipid membranes, proteins, and nucleic acids, which results in denaturation of cellular components or activation of the defense system in plants [39, 40].

Treatments of Fe_3_O_4,_ SiO_2_, and ZnO NPs at different concentrations induced oxidative stress in *Salvadora persica* callus by ROS production and lipid peroxidation that may in favor in BITC production. Both MDA and H_2_O_2_ are as reliable biomarkers of oxidative state. A significant increase in MDA levels was observed in *Salvadora persica* callus treated with all NPs with different concentrations compared with control, except 0.5 mg/l ZnO NPs. On the contrary, H_2_O_2_ levels significantly reduced in all NP-treated callus, except that treated with 2.0 mg/l Fe_3_O_4_ NPs. The imposed oxidative stress by different NPs can be alleviated by developing a defense mechanism including antioxidant scavenging enzymes such as POD and SOD to detoxify ROS induced by NPs. The oxidative enzymes SODs convert superoxide radicals into H_2_O_2_ and molecular oxygen, then peroxidases convert H_2_O_2_ into water. In plants, SODs contain several isoforms depending on their metal cofactor [40, 41]. The most prominent forms in *Salvadora persica* callus were one manganese SOD isoform (Mn SOD), two iron SOD isoforms (Fe SODI and II), and two copper/zinc isoforms (Cu/Zn SODI and II). The activity of Cu/Zn SOD I and II was increased in all Fe_3_O_4,_ SiO_2_, and ZnO NP treatments at different doses, reflecting the antioxidant activity of *Salvadora persica* callus in the response to NP stress, compared with control. As expected, the activity of the three POD isoforms was increased in all NP-treated callus compared with untreated callus. This may be a result of enhanced transcription of genes related to antioxidant capacity [17, 42]. These data are consistent with the results of an independent study, where Fe_3_O_4_ NPs (50 to 200 mg/g) initiate protective mechanisms to reduce oxidative stress by increased activities of POD and SOD in the wheat seedlings [43]. The ZnO NPs caused significant increases in SOD and POD activities in tomato plants to overcome the toxic effect of ZnO NPs [42]. Similar activity was demonstrated for SiO_2_ NPs effect on cotton plants [44].

The changes were observed in the ISSR profile indicating that the applied doses of NPs caused genetic variation that was dose and NP type-dependent. In the ISSR profile, the appearance of new DNA bands and the absence of normal ones could be described as a mutation, which probably results from DNA damage or rearrangements caused by NP-induced genetic variation. Genomic template stability is a reflection of the changes observed in the ISSR profile [16]. It was observed that the GTS percentage decreased with increased NP concentration for the three studied NPs. In accordance with previous literatures, Fe_3_O_4_ NPs displayed the lowest genetic toxicity in *Salvadora persica* callus. Previous studies also presented similar effects by the same NPs in flax callus cultures [36] and in rocket seedlings [33]. The concentration of 2 mg/l of both ZnO and SiO_2_ NPs strongly reduced GTS by 38.3 and 29.8%, respectively. Moreover, they scored the highest percentage of unique specific bands by 33.3 and 12.1%, respectively. The production of these bands may in favor of increased biomass for these treatments. Most likely, these bands are formed from the potential of NPs in causing genomic variation by impairing mitosis and altering DNA by inducing chromosomal anomalies [40].

The induced genetic variation of NPs can be explained by their interaction with DNA and/or nuclear proteins, following their diffusion into the cell, affecting the cell cycle, or a result of oxidative stress induced by reactive oxygen species and by affecting the ability of DNA repair mechanisms. The inverse relationship was demonstrated between the size of NPs and genotoxicity, while a direct relation was found with exposure duration and concentration [40].

Transmission electron microscope image analysis was performed to assess the cellular effect of different NPs on *Salvadora persica* callus. Images of TEM revealed that different NPs induced ultrastructure changes in *Salvadora persica* callus cells. Regular cellular organization and organelle structures have been observed in control cells. At a low concentration of 0.5 mg/l, TEM images showed the deposition of ZnO NPs in the callus cell wall via apoplastic course. At 2 mg/l concentration of all NPs, the cells appeared with thicker walls and the inflow of NPs inside the cytoplasm was much clear. These ultra-structural changes might be associated with oxidative stress induced by different NPs and a cause of their genotoxic effect. These results are in harmony with Radi et al. [45], who concluded that ZnO NPs mediated changes in cell ultrastructure of pomegranate callus cells. Also, Ghosh et al. [46] detected a similar effect of ZnO NPs in *Allium cepa* root cells. Slomberg and Schoenfisch [47] observed by TEM the deposition of SiO_2_ NPs into the roots of *Arabidopsis thaliana*. Yuan et al. [48] noticed the accumulation of Fe NPs in aggregates into cell walls and transported via the apoplastic pathway in the *Capsicum annuum* roots. In the current study, NPs used were 25–55 nm, which are larger than the estimated plant cell wall pore size of 5–20 nm. However, they have been observed in intercellular spaces. It is possible that NPs may induce cell wall deformation and increase its porosity to allow their access to intercellular space [48].

## Conclusion

The present study provides a direct approach for increasing biomass of *Salvadora persica* callus and BITC production by using NPs. An evidence for genetic variation and bioaccumulation of Fe_3_O_4_, SiO_2_, and ZnO NPs in *Salvadora persica* callus was provided, which shows the need for further research to overcome these side effects. In general, the results indicate that NPs have positive effects on *Salvadora persica* callus fresh weight and BITC accumulation, which encourage further studies on this point and using other more advanced in vitro methods to enhance the accumulation of this valuable anticancer compound, such as suspension cultures, bioreactors, and hairy root cultures.

## Data Availability

All data generated or analyzed during this study are included in this article.

## Abbreviations

BITC: Benzyl isothiocyanate
CFW: Callus fresh weight
GTS: Genomic DNA template stability
ISSR: Inter simple sequence repeats
MDA: *Malondialdehyde*
NBT: Nitro blue tetrazolium
NPs: Nanoparticles
PAGE: Polyacrylamide gel electrophoresis
POD: Peroxidase
RAPD: Random amplification of polymorphic DNA
ROS: Reactive oxygen species
SOD: Superoxide dismutases
TCA: Trichloroacetic acid
TBA: Thiobarbituric acid
TMB: 3,3′,5,5′-Tetramethylbenzidine
